# Involvement of Microglia in Retinal Ganglion Cell Injury Induced by IOP Elevation in a Rat Ex Vivo Acute Glaucoma Model

**DOI:** 10.3390/biomedicines13071670

**Published:** 2025-07-08

**Authors:** Taimu Sato, Makoto Ishikawa, Yukitoshi Izumi, Naoya Shibata, Kota Sato, Michiko Ohno-Oishi, Hiroshi Tawarayama, Hiroshi Kunikata, Charles F. Zorumski, Toru Nakazawa

**Affiliations:** 1 Department of Ophthalmology, Tohoku University Graduate School of Medicine, Sendai 980-8574, Japan; taimu.sato.d1@tohoku.ac.jp (T.S.); makoto.ishikawa.c2@tohoku.ac.jp (M.I.); kota.sato.c1@tohoku.ac.jp (K.S.); michiko.oishi.b4@tohoku.ac.jp (M.O.-O.); hiroshi.tawarayama.b4@tohoku.ac.jp (H.T.); hiroshi.kunikata.c7@tohoku.ac.jp (H.K.); 2 Ophthalmic Imaging and Information Analytics, Tohoku University Graduate School of Medicine, Sendai 980-8574, Japan; 3 Taylor Family Institute for Innovative Psychiatric Research, Washington University School of Medicine, St. Louis, MO 63110, USA; izumiy@wustl.edu (Y.I.); zorumskc@wustl.edu (C.F.Z.); 4 Center for Brain Research in Mood Disorders, Washington University School of Medicine, St. Louis, MO 63110, USA; 5 Department of Psychiatry, Washington University School of Medicine, St. Louis, MO 63110, USA; 6 Aoba Eye Clinic, Akita 010-1413, Japan; htm4352@yahoo.co.jp; 7 Department of Advanced Ophthalmic Medicine, Tohoku University Graduate School of Medicine, Sendai 980-8574, Japan; 8 Department of Retinal Disease Control, Tohoku University Graduate School of Medicine, Sendai 980-8574, Japan

**Keywords:** glaucoma, intraocular pressure, neuroinflammation, NLRP3 inflammasome, interleukin-1β, neuroprotection, PLX5622

## Abstract

**Background**: An acute angle-closure attack (AAC) is an ocular emergency that results from a rapid increase in intraocular pressure (IOP). Sustained IOP elevation induces severe degeneration of retinal ganglion cells (RGCs) without treatment. Overactivated microglia, key participants in innate immune responses, have critical roles in the pathogenesis of IOP-induced RGC death, although precise mechanisms remain unclear. In the present study, we used a rat ex vivo acute glaucoma model to investigate the role of microglial signaling in RGC death and examined whether pharmacological depletion of microglia using a CSF-1R inhibitor, PLX5622, exerts neuroprotection against pressure-induced retinal injury. **Methods**: Ex vivo rat retinas were exposed to hydrostatic pressure (10 mmHg or 75 mmHg) for 24 h. Pressure-dependent changes in retinal microglia and RGCs were detected by immunofluorescence. Morphological changes in the retina and RGC apoptosis were examined using light microscopy and TUNEL staining, respectively. The expression of NLRP3, active caspase-1, pro IL-1β, and IL-1β were examined using Western blotting. Effects of PLX5622, an agent that depletes microglia, were examined in morphology, apoptosis, and protein expression assays, while TAK-242, a TLR4 inhibitor, was examined against protein expression. **Results**: Pressure loading at 75 mmHg markedly increased activated microglia and apoptotic RGCs in the isolated retinas. Western blotting revealed increases in expression of NLRP3, active caspase-1, pro IL-1β, and IL-1β at 75 mmHg compared to 10 mmHg. Inhibition of pressure-induced increases in NLRP3 by TAK-242 indicates that pressure elevation induces RGC death via activation of the TLR4–NLRP3 inflammasome cascade. PLX5622 depleted microglia at 75 mmHg and significantly decreased expression of NLRP3, active caspase-1, pro IL-1β, and IL-1β at 75 mmHg, resulting in preservation of RGCs. **Conclusions**: These results indicate that pressure elevation induces proliferation of inflammatory microglia and promotes IL-1β production via activation of the TLR4–NLRP3 inflammasome cascade, resulting in RGC death. Pharmacological depletion of microglia with PLX5622 could be a potential neuroprotective approach to preserve RGCs from inflammatory cytokines in AAC eyes.

## 1. Introduction

Glaucoma is one of the leading causes of irreversible blindness [1] and is characterized by selective degeneration of retinal ganglion cells (RGCs) [2]. Among all types of glaucoma, acute angle-closure attack (AAC) has the highest rate of irreversible blindness [3]. The AAC is characterized by sudden increase in IOP that can reach 80 mmHg [4] and cause severe visual disturbance without treatment [3,4,5,6]. Although the mechanisms underlying pressure-induced RGC injury in AAC remain unclear, accumulating evidence suggests its crucial role for activation of retinal innate immune responses [7,8].

Microglia are the principal innate immune cells of myeloid origin in the nervous system and play important roles in glaucomatous RGC death [9,10,11,12]. Overactivation of microglia can result in the production of proinflammatory cytokines, including interleukin (IL)-1β [13,14], and promote the progression of glaucoma [14].

The nucleotide-binding and oligomerization domain, leucine-rich repeat-, and pyrin domain-containing protein 3 (NLRP3) and IL-1β inflammasome cascade is a critical mechanism in microglia activation [15]. The NLRP3 inflammasome is a typical NOD-like receptor (NLR), composed of an NLRP3 sensor, apoptosis-associated speck-like protein containing a caspase-recruitment domain, and the effector protein caspase-1. Once activated, the NLRP3 inflammasome mediates the cleavage of pro-caspase-1 into active caspase-1, leading to the maturation of pro-IL-1β [16].

Microglia express all classes of innate immune signaling receptors, also called pattern recognition receptors (PRRs). Toll-like receptors (TLRs) are key PRRs in the initiation of the innate immune response [17,18,19]. TLR4, in particular, has been shown to have a central role in retinal ischemia/reperfusion (I/R) injuries [20]. Neuronal death following ischemic injury activates intense neuroinflammation, which triggers TLR4 signaling and downstream cascades resulting in NLRP3 inflammasome activation and IL-1β production. Furthermore, TLR4-deficient mice are protected against ischemic brain damage [21,22]. The TLR4–NLRP3 inflammasome cascade may also play a major role in ocular pressure-dependent neuropathy caused by microglia. If reactive microglia contribute to pressure-induced retinal injury in AAC, pharmacological inhibition or depletion of microglia might be promising approaches to promote neuroprotection in acute glaucoma [23,24].

In the present study, we used an ex vivo rat glaucoma model [25] to investigate the microglial signaling pathway mediating RGC death and describe key roles of TLR4 and IL-1β. In addition, we examined whether pharmacological suppression of microglia can promote neuroprotection against pressure-induced retinal injury.

## 2. Materials and Methods

All animal experiments in this study complied with the Association for Research in Vision and Ophthalmology (ARVO) statement for the use of animals in ophthalmic and vision research. The study design and all methods involved in the experiments were approved by the Institutional Animal Care and Use Committee at Tohoku University in accordance with the Research Animal Guidelines (approval number 2022-055).

### 2.1. Rat Ex Vivo Eyecup Preparation

Thirty–thirty-seven-day-old male Sprague Dawley (SD) rats were purchased from SLC (Shizuoka, Japan). All SD rats were maintained at the Tohoku University Graduate School of Medicine under a 12 h light/dark cycle. After the anterior half of the eye was excised, the posterior cup was placed in the bottom of a 100 mL glass beaker filled with (in mM) aCSF (artificial cerebrospinal fluid) containing 124 NaCl, 5 KCl, 2 MgSO_4_, 2 CaCl_2_, 1.25 NaH_2_PO_4_, 22 NaHCO_3_, and 10 glucose, and a sealed pressure loading system and incubated at 30 °C for 24 h [25] (also see Supporting Figure S1). A 95% O_2_-5% CO_2_ gas mixture was delivered into the incubation buffer.

In some experiments, a potent and selective TLR4 signaling inhibitor, TAK-242 (Cat#13871, Cayman Chemical, Ann Arbor, MI, USA) (1 μM), or a CSF-1R inhibitor, PLX5622 (Cat#HY-114153, MedChemExpress, Monmouth Junction, NJ, USA) (10 μM or 20 μM), was administered in aCSF at the time of experiment. After pressure loading at 75 mmHg for 24 h at 30 °C, the pressure inside the chamber was gradually reduced to atmospheric pressure (0 mmHg) over a 1 min period to avoid abrupt decompression.

### 2.2. Whole Mounted Retinas and Immunostaining

After incubation, eye cups were fixed with 4% paraformaldehyde—0.1 M phosphate—overnight at 4 °C. Retinas were then detached from the pigment epithelium and flat-mounted. After rinse with PBS, retinas were incubated with 1.5% goat serum albumin in Tagmentation DNA buffer containing 1% Triton X-100 and 1% dimethyl sulfoxide by gently shaking at room temperature for 1 h. After rinse with PBS, retinas were incubated with rabbit anti-RNA binding protein, mRNA processing factor (RBPMS) antibody [26] (1:1000, Cat#ab152101, Abcam, Waltham, MA, USA), or rabbit anti-Iba-1 antibody solution (1:500, Cat#019-19741, FUJIFILM Wako Pure Chemical, Osaka, Japan) by gently shaking at 4 °C, overnight. After rinsing 3 times using PBS, retinas were incubated with goat anti-rabbit IgG (H&L) cross-adsorbed secondary antibody, Alexa Fluor™ 488 (1:1000, Cat#A-11008, Invitrogen Corp, Carlsbad, CA, USA) at 4 °C, overnight. After rinsing 3 times with PBS, retinas were stretched on glass slides using Vectashield Mounting Medium (Cat#H-1000, Vector Laboratories Inc., Newark, CA, USA). Retinal flat-mount preparations were imaged in each of the four defined retinal quadrants using a confocal microscope (Ti-E, Nikon, Shinagawa, Japan), as previously described [25]. Density of RBPMS-positive RGCs and Iba-1-positive microglia was calculated by measurement of four different quadrants using Image J software (version 1.37, image age analysis software developed by Wayne Rasband at NIH, Bethesda, MD, USA), and the density per square millimeter was measured and compared in experimental retinas.

### 2.3. Microglial Sholl Analysis

To evaluate the morphological complexity of retinal microglia, we performed Sholl analysis on Iba-1-stained flat-mounted retinas, prepared as described in Section 2.2. Retinal images were acquired using a confocal microscope (Ti-E, Nikon, Shinagawa, Japan) at 20× magnification. For each retina, three representative fields were randomly selected from different retinal quadrants, and three well-defined microglial cells were manually chosen per field, excluding overlapping, poorly stained, or ambiguously shaped cells. Only isolated microglia exhibiting clearly identifiable somata and intact, radiating processes were selected for analysis. According to previously described methods [27], a series of concentric circles centered on the soma of each microglial cell were drawn at 5 μm intervals, ranging from 5 μm to 100 μm in radius. The number of intersections between microglial processes and each circle was manually counted by visual inspection. For each retina, the total number of intersections was calculated by summing the values from nine cells (three cells per field across three fields). In addition, the number of intersections at each concentric radius was recorded to evaluate radial process distribution. All image selection and intersection counting were performed by an observer unaware of experimental conditions.

### 2.4. Microglial Soma Area Measurement

Microglial soma area was measured using ImageJ (version 1.37) from the same Iba-1-stained flat-mounted retinal images used for Sholl analysis. For each retina, three representative fields were analyzed, and three well-isolated microglial cells per field were selected. Binary images were generated by manual thresholding, and soma regions were outlined using the Freehand Selection tool available in ImageJ. The area of each ROI was measured after clearing the surrounding background. The spatial scale was set to 0.8 μm/pixel, and results were expressed in μm^2^. All measurements were performed by a single blinded observer.

### 2.5. Iba-1 and CD68 Double Immunofluorescence on Cryosections

Cryosections were prepared from retinas fixed with 2% paraformaldehyde for 30 min. After fixation, the sections were washed three times with Tween-PBS (Tw-PBS) for 5 min each, then permeabilized in 0.5% Triton X-100 in Tw-PBS for 10 min at room temperature, followed by three additional 5 min washes in Tw-PBS. Non-specific binding was blocked by incubation with 10% donkey serum in Tw-PBS for 1 h at room temperature.

We examined whether pressure elevation promotes a shift toward M1-type activation by performing double immunofluorescence staining of retinal cryosections for Iba-1 and CD68 (M1 marker) [28]. The sections were then incubated overnight at 4 °C with a mixture of primary antibodies: rabbit anti-CD68 (1:400, Cat#25747-1-AP, Proteintech, Rosemont, IL, USA) and goat anti-Iba1 (1:200, Cat#011-27991, FUJIFILM Wako Pure Chemical Corporation, Osaka, Japan), both diluted in Tw-PBS containing 1% donkey serum. After three washes in Tw-PBS, the sections were incubated at room temperature for 1 h with secondary antibodies: biotinylated donkey anti-rabbit IgG (1:1000, Cat#711-165-152, Jackson ImmunoResearch, West Grove, PA, USA) and Cy3 AffiniPure donkey anti-goat IgG (1:500, Cat#705-165-003, Jackson ImmunoResearch, West Grove, PA, USA). After another series of three 5 min washes with Tw-PBS, the sections were incubated with streptavidin conjugated to Alexa Fluor™ 647 (1:1000, Cat#S12374, Invitrogen Corp, Carlsbad, CA, USA) for 2 h at room temperature. Following a final wash step, the sections were mounted with Vectashield Mounting Medium containing DAPI (Cat#H-1200, Vector Laboratories Inc., Newark, CA, USA). Fluorescence images were acquired using a fluorescence microscope (BZ-800X, Keyence, Osaka, Japan).

### 2.6. Apoptosis

To visualize apoptotic cells, we used the ApopTag Fluorescein In Situ Apoptosis Detection Kit (Sigma-Aldrich, St. Louis, MO, USA) according to the manufacturer’s instructions. Nuclei were counterstained with Vectashield Mounting Medium with DAPI (Cat#H-1200, Vector Laboratories). Apoptotic cells were visualized and imaged using a fluorescence microscope (BZ-800X, Keyence, Osaka, Japan). After the length of each retinal section was measured using NIH ImageJ software (version 1.37), cells in the total length of the section were counted, and cell density was calculated per 1000 μm retinal section. Semi-thin retinal sections were examined and photographed using a fluorescence microscope (BZ-800X, Keyence, Osaka, Japan).

A number of cryosections were subjected to TUNEL staining using the ApopTag Fluorescein In Situ Apoptosis Detection Kit (Sigma-Aldrich, St. Louis, MO, USA) according to the manufacturer’s instructions. After staining, the sections were washed three times with Tween-PBS (Tw-PBS) for 5 min each. The sections were then incubated in 0.5% Triton X-100 in Tw-PBS for 10 min at room temperature, followed by three additional washes in Tw-PBS. Non-specific binding was blocked by incubation with 10% donkey serum in Tw-PBS for 1 h at room temperature. After blocking, the sections were incubated overnight at 4 °C with rabbit anti-RBPMS antibody (1:1000, Cat#ab152101, Abcam, Waltham, MA, USA). On the following day, sections were washed three times with Tw-PBS and incubated for 1 h at room temperature with goat anti-rabbit IgG (H&L) cross-adsorbed secondary antibody conjugated to Alexa Fluor™ 488 (1:1000, Cat#A-11008, Invitrogen Corp, Carlsbad, CA, USA). After a final series of washes in Tw-PBS (three times, 5 min each), the sections were mounted with Vectashield Mounting Medium containing DAPI (Cat#H-1200, Vector Laboratories Inc., Newark, CA, USA). Fluorescent images were acquired using a fluorescence microscope (BZ-800X, Keyence, Osaka, Japan).

### 2.7. Preparation of Materials for Light Microscopy

After each experiment was completed, retinal specimens were immersion-fixed overnight at 4 °C in 1% paraformaldehyde and 1.5% glutaraldehyde in 0.1 M phosphate buffer. Subsequently, the specimens were postfixed in 1% osmium tetroxide and 0.1 M phosphate buffer for 60 min. Postfixed retinas were dehydrated in graded ethanol, embedded in Epon 812 (TAB Laboratories, Aldermaston, UK), and sectioned into 1 μm thick sections. These semi-thin sections were stained with toluidine blue and observed under an optical microscope (BZ-800X, Keyence, Osaka, Japan).

### 2.8. Data Analysis of Morphometrics

The thickness of the optic nerve fiber layer (NFLT) was measured at a distance of 1200 μm from the center of the optic disk, according to previously published methods [25]. The extent of neuronal damage was scored under an optical microscope using the Neuronal Damage Score (NDS), which rates neuronal damage in the inner nuclear layer (INL) and inner plexiform layer (IPL) on a scale of 0 to 3, with 0 indicating no neuronal damage and 3 indicating very severe damage.

### 2.9. Western Blot Analysis

After each experiment was completed, the retina was carefully and gently detached using a surgical blade and thin forceps. The separated retina was frozen at −80 °C. Retinas were then homogenized in 1% Protease inhibitor/RIPAbuffer (Cat#78425, Thermo Fisher scientific Inc., Waltham, MA, USA). The tissue extracts were ultrasonicated and clarified by centrifugation at 15,000× *g* for 15 min at 4 °C. The protein concentrations in supernatants were assayed (Pierce BCA Protein Assay Kit, Cat#23227, Thermo Fisher Scientific Inc). Fifty micrograms of retinal extract were subjected to SDS polyacrylamide gel electrophoretic analysis using 10% Mini-PROTEAN TGX Precast Protein Gels (Cat#4561036, Bio-Rad, Hercules, CA, USA) for NLRP3 and 12% Mini-PROTEAN TGX Precast Protein Gels (Cat#4561046, Bio-Rad) for IL-1β and caspase-1. The proteins were then transferred to Trans-Blot Turbo Mini 0.2 µm PVDF Transfer Packs (Cat#1704156, Bio-Rad). After blocking in 5% skim milk for 1 h at room temperature, PVDF membranes were incubated with anti-NLRP3 antibody (1:1000, Cat#MA5-32255, Invitrogen Corp), anti-pro IL-1β antibody (0.25 µg/mL, Cat#AF-401-NA, R&D Systems, Minneapolis, MN, USA), anti-IL-1β antibody (1:1000, Cat#A17361, ABclonal Technology Inc., Woburn, MA, USA), or anti-caspase-1 antibody (1:500, Cat#A0964, ABclonal Technology Inc., Woburn, MA, USA) at 4 °C, overnight. After washing with T-PBS three times each for 5 min, PVDF membranes were incubated with donkey anti-rabbit IgG (H + L) (1:100,000, Cat#711-035-152, Jackson ImmunoResearch Laboratories) for NLRP3, IL-1β, and caspase-1, and with donkey anti-goat IgG (H + L) (1:100,000, Cat#705-035-003, Jackson ImmunoResearch Laboratories) for pro IL-1β at room temperature for 1 h. Immunoblots were visualized using ImmunoStar LD (Cat#292-69903, FUJIFILM Wako Pure Chemical, Osaka, Japan), and photographed using ChemiDoc XRS+ (Bio-Rad) adjusted to avoid over- or undersaturation. The membranes were then stripped and reprobed with β-actin using anti-mouse β-actin monoclonal antibody (1:5000, Cat#A5316, Sigma-Aldrich, Burlington, MA, USA), followed by incubation with donkey anti-mouse IgG (H + L) (1:100,000, Cat#715-035-150, Jackson ImmunoResearch Laboratories) at room temperature for 1 h to ensure that protein loadings were equal. Western blot bands were analyzed using Image J. The relative grayscale values of the obtained bands were calculated with respect to the β-actin band of each pressure. Western blotting for each target protein (e.g., NLRP3, IL-1β, pro IL-1β, caspase-1) was performed using separate gels and on different experimental days. In some cases, even for the same target protein, experimental samples and controls were not run on the same gel or on the same day. To ensure comparability, all blots were processed using the same protocols and reagents, and quantification was performed under standardized conditions. This limitation is acknowledged and was taken into account in data interpretation.

### 2.10. Image Processing

All images were processed using Adobe Photoshop 2025 (version 26.2.0; Adobe Inc., San Jose, CA, USA). Only linear adjustments to brightness and contrast were applied uniformly across the entire image. No region-specific enhancements or alterations were made. The adjusted images accurately reflect the original data.

### 2.11. Statistical Analysis

The primary outcome measure was the density of RBPMS-positive RGCs, as this directly reflects the extent of retinal neurodegeneration. Other outcomes, including Iba-1-positive microglia counts, inflammasome-related protein expression, and morphological changes, were treated as secondary measures to support mechanistic interpretation.

T.S. performed the analyses using the free statistical software R (version 4.3.3; The R Foundation for Statistical Computing, Vienna, Austria) on a personal computer. Descriptive statistical results are presented using the mean values (mean) ± standard deviation (SD). For comparison with both the control and other conditions, we used t-tests, and Tukey’s multiple comparison test after one-way ANOVA. For all analyses, *p* values were considered statistically significant when the values were less than 0.05 (two-tailed).

Animals were randomly assigned to the 10 mmHg or 75 mmHg pressure groups from the same cage using a random allocation method to minimize biological variability and environmental bias. Analyses of retinal ganglion cells, microglia, apoptotic cells, nerve fiber layer thickness, and neuronal damage score were conducted by observers blinded to the experimental groups. Blinding was maintained throughout image acquisition and quantitative assessment to avoid observer bias.

The sample size for each group (*n* = 4–8) was determined based on prior ex vivo studies using similar retinal preparations. This size was considered adequate to detect significant differences in key outcome measures, including RGC survival, protein expression levels, and cell death, while accounting for biological variability.

To minimize confounding factors, all procedures were conducted in parallel using identical handling protocols and timing. Experimental conditions, including temperature, incubation period, and solution preparation, were maintained consistently across all groups.

## 3. Results

### 3.1. Pressure-Mediated Microglial Proliferation and Retinal Degeneration in an Ex Vivo Model

**Figure 1:**
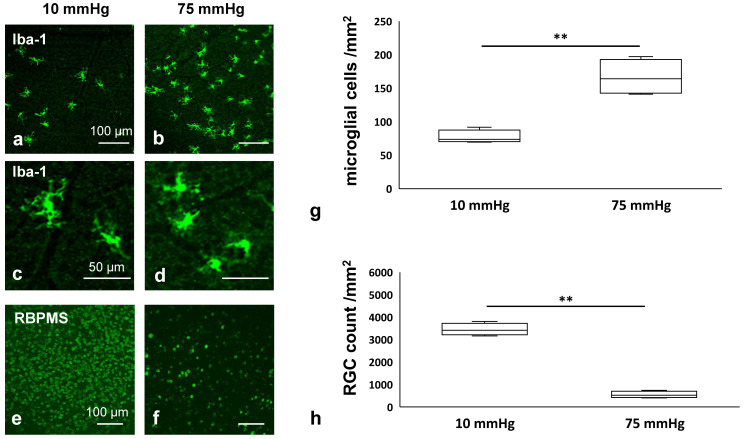
Pressure-induced morphological changes in microglia and RGC survival in whole mounted retinas. (**a**,**b**) Iba-1 positive microglia in pressure-loaded whole mounted retinas ((**a**) 10 mmHg, (**b**) 75 mmHg). (**c**,**d**) High magnification images of Iba-1-positive microglia at 10 mmHg (**c**) and 75 mmHg (**d**). (**e**,**f**) RGC survival in pressure loaded whole mounted retinas immunostained with an anti-RBPMS antibody, a marker of RGCs ((**e**) 10 mmHg, (**f**) 75 mmHg). (**g**) The density of Iba-1-positive microglia was significantly increased at 75 mmHg compared to 10 mmHg (*n* = 4 in each experiment, Student’s *t*-test, ** *p* < 0.01). (**h**) RBPMS-positive RGCs decreased at 75 mmHg compared to 10 mmHg (*n* = 5 in each experiment, Student’s *t*-test, ** *p* < 0.01).

We initially examined the effects of pressure elevation on microglial density and morphology in whole mounted retinas immunolabeled with anti-Iba-1 antibody, a microglia-specific marker. Figure 1a demonstrates confocal images of Iba-1-labeled microglia of a control eye incubated at 10 mmHg. Following incubation of retinas under elevated pressure (75 mmHg), there was a clear increase in the density of Iba-1-positive cells (Figure 1b). The morphology of microglia at 10 mmHg was ‘ramified’ with resting-type cell bodies and elongated dendrites (Figure 1c). At 75 mmHg, microglia showed significant morphological changes with retracted processes and hypertrophic cell bodies, indicating microglial activation (Figure 1d).

We next examined the effects of pressure elevation on RGCs in whole mounted retinas immunolabeled with an anti-RBPMS antibody, an RGC-specific marker. Figure 1e shows confocal images of RBPMS-labeled RGCs of a control eye incubated at 10 mmHg. As previously reported [25], we observed a reduction in RBPMS-positive cells following incubation at elevated pressure (75 mmHg) (Figure 1f). Figure 1g and 1h show the density of Iba-1-positive microglia and RBPMS-positive RGCs in the retina in the two conditions, respectively. Quantitative analysis showed that the density of Iba-1-positive microglia was significantly increased from 77.1 ± 8.6 cells/mm^2^ at 10 mmHg to 166.6 ± 23.1 cells/mm^2^ at 75 mmHg. In contrast, the density of RBPMS-positive RGCs significantly decreased from 3370.6 ± 260.4 cells/mm^2^ at 10 mmHg to 535.0 ± 115.0 cells/mm^2^ at 75 mmHg.

### 3.2. Changes in Markers Associated with NLRP3 Inflammasome Pathway

We examined the involvement of NLRP3 Inflammasome pathway in pressure-induced retinal injury by Western blot analysis of NLRP3, IL-1β, and pro IL-1β. NLRP3 protein plays a critical role in the NLRP3 inflammasome pathway. IL-1β is an effector of the NLRP3 inflammasome pathway, and pro IL-1β is its precursor form. It is well known that in Western blotting bands may appear at unexpected molecular weights due to antibody cross-reactivity with off-target proteins, proteolytic degradation of the target protein, or the formation of protein oligomers [29,30]. In the present study, several bands deviating markedly from the theoretical molecular weights of NLRP3 and pro–IL-1β were observed. As these bands did not correspond to any known isoforms or cleavage products of the respective proteins, they were interpreted as non-specific signals resulting from antibody cross-reactivity. Based on molecular weight, we identified specific bands for NLRP3, IL-1β, and pro IL-1β at approximately 110 kDa, 17 kDa, and 37 kDa, respectively.

**Figure 2:**
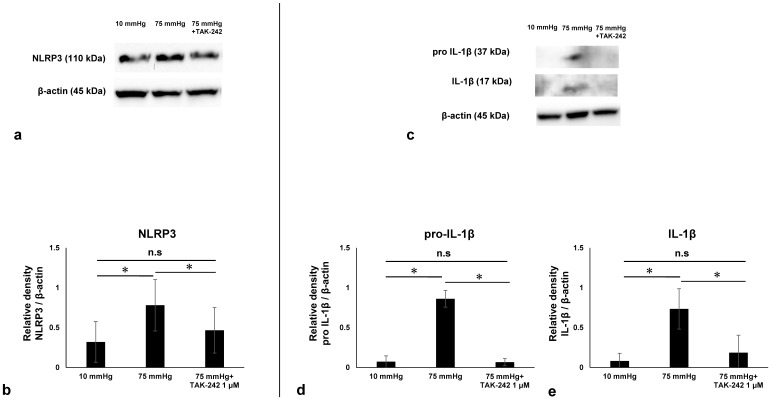
Western blotting of pressure-dependent changes in NLRP3, pro IL-1β, and IL-1β. (**a**). Representative immunoblotting for NLRP3 and actin. (**b**). Relative densitometry analysis of NLRP3 expression (*n* = 6 in each experiment, Tukey’s multiple comparison test, * *p* < 0.05). (**c**). Representative immunoblotting for pro IL-1β, IL-1β, and actin. (**d**). Relative densitometry analysis of pro IL-1β (*n* = 6 in each experiment, Tukey’s multiple comparison test, * *p* < 0.05). (**e**). Relative densitometry analysis of IL-1β expression (*n* = 6 in each experiment, Tukey’s multiple comparison test, * *p* < 0.05). The samples shown were derived from separate gels and experiments conducted on different days. All blots were processed using identical protocols. “n.s”: non-significant.

To determine the potential role of the PRR, TLR4, in pressure-induced increases in NLRP3, we examined the effects of the potent and selective TLR4 signaling inhibitor, TAK-242. Administration of TAK-242 inhibited pressure-induced increases in NLRP3 (Figure 2a,b), IL-1β, and pro IL-1β (Figure 2c–e), indicating that the TLR4–NLRP3 inflammasome axis was activated in this acute glaucoma model.

Quantitative Western Blot analysis demonstrated that pressure elevation significantly increased expression of NLRP3 (Figure 2a,b). Quantitative Western Blot analysis also demonstrated that pressure elevation significantly increased expression of IL-1β and pro IL-1β (Figure 2). Figure 2d,e show the relative density of IL-1β and pro IL-1β in retinas under each condition. Quantitative analysis showed that NLRP3 expression increased from 0.25 ± 0.06 at 10 mmHg to 1.06 ± 0.15 at 75 mmHg and was reduced to 0.39 ± 0.11 with TAK-242 treatment (Figure 2b). Similarly, pro IL-1β levels increased from 0.073 ± 0.072 at 10 mmHg to 0.86 ± 0.10 at 75 mmHg and decreased to 0.067 ± 0.046 with TAK-242 (Figure 2d). Mature IL-1β expression increased from 0.081 ± 0.097 at 10 mmHg to 0.73 ± 0.25 at 75 mmHg and was suppressed to 0.19 ± 0.22 with TAK-242 treatment (Figure 2e).

### 3.3. PLX5622 Prevents Pressure-Induced Microglial Proliferation

**Figure 3:**
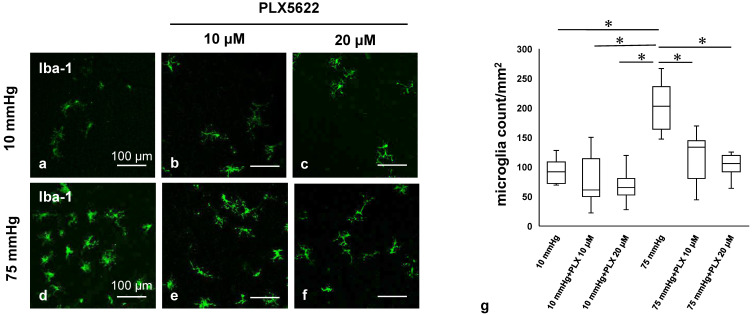
Pharmacological depletion of microglia using PLX5622. (**a**–**c**) PLX5622-dependent microglial distribution at 10 mmHg: (**a**) without PLX5622, (**b**) 10 μM PLX5622, (**c**) 20 μM PLX5622. (**d**–**f**) PLX5622-dependent microglial distribution at 75 mmHg: (**d**) without PLX5622, (**e**) 10 μM PLX5622, (**f**) 20 μM PLX5622. (**g**) The density of immune-positive microglia for Iba-1 antibody in whole mounted retinas under each condition (*n* = 7 in each experiment, Tukey’s multiple comparison test, * *p* < 0.05).

To determine more directly the role of microglia in the effects we observed in experiments outlined above, we used the colony stimulating factor receptor 1 inhibitor, PLX5622, an agent that depletes microglia selectively [24]. We initially examined the pharmacological effects of PLX5622 by measuring changes in the density of microglia in whole mounted retinas at control and elevated pressures using the anti-Iba-1 antibody as a microglial marker. Figure 3a demonstrates confocal images of Iba-1-labeled microglia in ex vivo eye cups incubated without PLX5622 at 10 mmHg. Administration of 10 μM (Figure 3b) or 20 μM PLX5622 (Figure 3c) showed no significant changes in Iba-1 staining at 10 mmHg. Figure 3d demonstrates increased Iba-1-labeled microglia in ex vivo eye cups incubated without PLX5622 at 75 mmHg. Administration of 10 μM (Figure 3e) or 20 μM PLX5622 (Figure 3f) resulted in significant decreases in Iba-1 staining at 75 mmHg. Figure 3g shows the density of Iba-1-positive microglia in the retina in each condition. Quantitative analysis showed that the density of Iba-1-positive microglia increased from 92.9 ± 20.4 cells/mm^2^ at 10 mmHg to 200.4 ± 38.0 cells/mm^2^ at 75 mmHg. Administration of PLX5622 reduced microglial density at high pressure to 115.9 ± 39.5 cells/mm^2^ with 10 μM and 101.6 ± 19.1 cells/mm^2^ with 20 μM PLX5622. Under normo-tensive conditions, microglial density was 81.0 ± 42.1 cells/mm^2^ and 60.7 ± 17.5 cells/mm^2^ with 10 μM and 20 μM PLX5622, respectively (Figure 3g).

### 3.4. Microglial Morphology Assessed by Sholl Analysis and Soma Area Measurement

While 10 μM and 20 μM PLX5622 had similar effects in the preceding experiments, we subsequently focused on 20 μM PLX5622 because of its more consistent ability to alter the effects of high pressure. To determine whether the morphological changes observed in Iba-1-positive microglia (Figure 1c,d) and the reduction in their density following PLX5622 treatment (Figure 3) reflected microglial activation, we performed Sholl analysis and soma area measurements on flat-mounted retinas.

**Figure 4:**
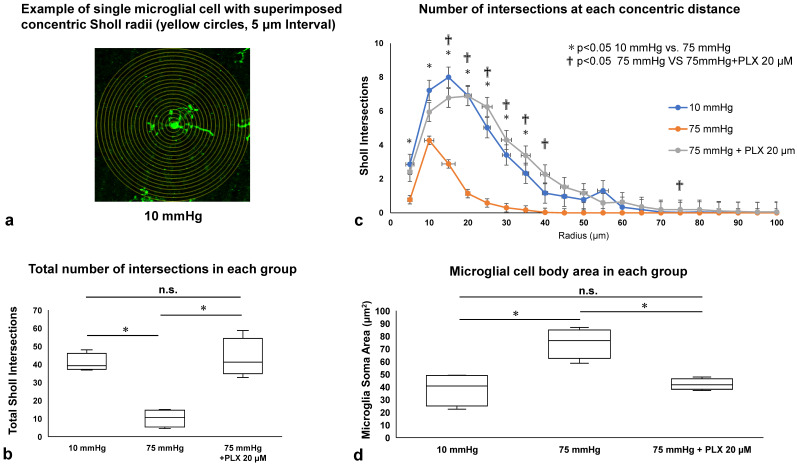
Sholl analysis and soma area of microglia. (**a**) Tracing of Iba-1 labeled microglia at 10 mmHg for Sholl analysis. (**b**) Total number of Sholl intersections under each condition (*n* = 4 per group, Tukey’s multiple comparison test, * *p* < 0.05). (**c**) Mean distribution of Sholl intersections as a function of distance from the microglial soma (*n* = 4 per group, Tukey’s multiple comparison test, * *p* < 0.05 for 10 mmHg vs. 75 mmHg; † *p* < 0.05 for 75 mmHg vs. 75 mmHg + PLX 20 μM). (**d**) Mean soma area of microglia under each condition (*n* = 4 per group, Tukey’s multiple comparison test, * *p* < 0.05). “n.s.”: non-significant.

The total number of Sholl intersections, an indicator of dendritic complexity, was significantly decreased at 75 mmHg (10.17 ± 4.22) compared with 10 mmHg (40.81 ± 4.31, * *p* < 0.05). Treatment with 20 μM PLX5622 under 75 mmHg significantly increased the number of intersections (43.42 ± 9.46), restoring it to a level comparable to 10 mmHg (* *p* < 0.05 vs. 75 mmHg) (Figure 4b).

We next analyzed the distribution of intersections as a function of distance from the soma. At 75 mmHg, the number of intersections was significantly reduced between 5 and 35 μm from the soma compared with 10 mmHg (* *p* < 0.05). This reduction was not observed in the 75 mmHg + PLX5622 group, where the number of intersections at each distance remained comparable to the 10 mmHg group (Figure 4c).

To further assess microglial morphology, we measured the soma area of Iba-1–positive cells. Microglial soma area was significantly larger at 75 mmHg (74.56 ± 10.32 μm^2^) than at 10 mmHg (38.19 ± 11.24 μm^2^, * *p* < 0.05). This enlargement was markedly attenuated by PLX5622 treatment (42.04 ± 3.77 μm^2^), resulting in soma areas comparable to those observed at 10 mmHg (* *p* < 0.05 vs. 75 mmHg) (Figure 4d).

Collectively, these findings indicate that microglia at 10 mmHg maintain a ramified morphology characterized by a small soma and multiple long, branched processes. In contrast, microglia at 75 mmHg adopt an amoeboid shape with enlarged soma and reduced process complexity, indicative of activation. PLX5622 treatment preserved the ramified morphology, suggesting suppression of pressure-induced microglial activation.

### 3.5. CD68 Expression Among Iba-1-Positive Microglia

**Figure 5:**
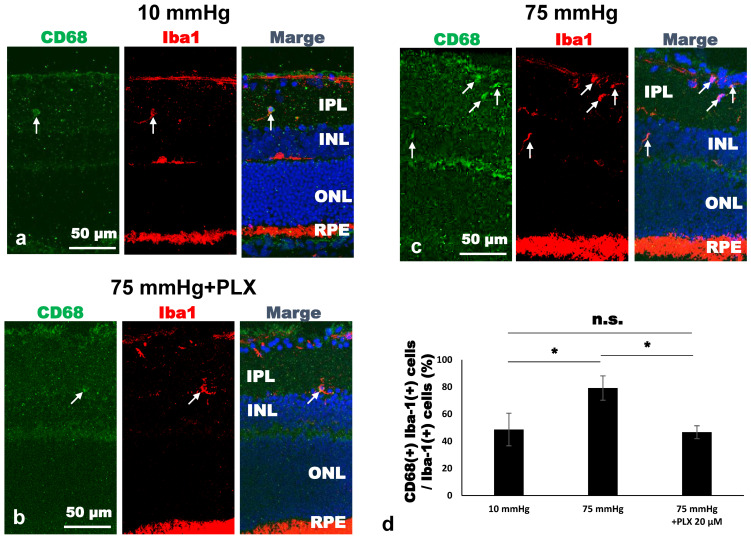
Cryosections double-stained with anti-CD68 antibody (green) and anti-Iba-1 antibody (red). Co-localization of CD68/Iba-1 is visualized in a merged image. (**a**) At 10 mmHg, there were a small number of CD68-positive/Iba-1-positive microglia. (**b**) At 75 mmHg, microglia proliferated, and the proportion of CD68-positive microglia increased. (**c**) An increase in CD68-positive microglia was attenuated by treatment with 20 μM PLX5622, restoring the proportion to levels comparable to those at 10 mmHg. (**d**) Quantification of the proportion of CD68-positive cells among Iba-1-positive microglia under each condition (*n* = 3 per group, Tukey’s multiple comparison test, * *p* < 0.05). “n.s.”: non-significant.

As shown in Figure 4, microglia exhibited morphological features of activation at 75 mmHg. Since activated microglia are broadly categorized into cytotoxic M1 and neuroprotective M2 phenotypes, we examined whether pressure elevation promotes a shift toward M1-type activation by performing double immunofluorescence staining of retinal cryosections for Iba-1 and CD68. Representative images are shown in Figure 5a–c. Quantitative analysis demonstrated that the proportion of CD68-positive cells among Iba-1–positive microglia significantly increased at 75 mmHg (79.17 ± 9.00%) compared with 10 mmHg (48.61 ± 11.95%, * *p* < 0.05). This increase was markedly suppressed by treatment with 20 μM PLX5622 (46.67 ± 4.71%), restoring the proportion to a level comparable to that observed under 10 mmHg (* *p* < 0.05) (Figure 5d).

These findings, together with the microglial density data in Figure 3, suggest that elevated pressure enhances both the number and proportion of cytotoxic M1-type microglia, whereas PLX5622 treatment attenuates this shift and preserves a microglial profile similar to the control condition.

### 3.6. PLX5622 Preserves the RNA-Binding Protein (RBPMS) Under High Pressure

**Figure 6:**
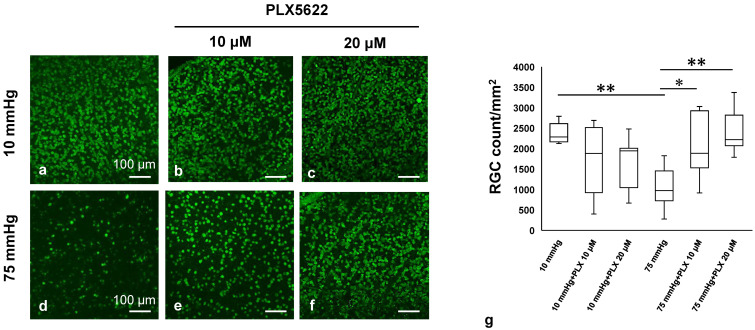
(**a**–**c**) PLX5622-dependent RGC distribution at 10 mmHg ((**a**) without PLX5622, (**b**) 10 μM PLX5622, (**c**) 20 μM PLX5622). (**d**–**f**) PLX5622-dependent RGC distribution at 75 mmHg ((**d**) without PLX5622, (**e**) 10 μM PLX5622, (**f**) 20 μM PLX5622). (**g**) RGC survival in the whole mount retina under each condition (*n* = 8 in each experiment, Tukey’s multiple comparison test, * *p* < 0.05, ** *p* < 0.01).

We examined neuroprotective effects of PLX5622 in whole mounted retina immunolabeled with the anti-RBPMS antibody to monitor changes in RGCs. Figure 6a demonstrates confocal images of RBPMS-labeled RGCs of a control eye incubated at 10 mmHg. Administration of 10 μM (Figure 6b) or 20 μM PLX5622 (Figure 6c) did not show a significant difference in RBPMS staining at 10 mmHg. In contrast, the reduction in positive cells for RBPMS by pressure elevation (75 mmHg) (Figure 6d) was attenuated by both 10 μM (Figure 6e) and 20 μM PLX5622 (Figure 6f). Figure 6g shows the density of RBPMS-positive RGCs in the retina in each condition. Quantitative analysis showed that the density of RBPMS-positive RGCs was 2383.3 ± 229.1 cells/mm^2^ at 10 mmHg and decreased to 1030.1 ± 461.6 cells/mm^2^ at 75 mmHg. PLX5622 treatment preserved RGC density to 2082.4 ± 721.4 cells/mm^2^ at 75 mmHg with 10 μM and 2392.9 ± 487.0 cells/mm^2^ with 20 μM. Under 10 mmHg conditions, PLX5622 at 10 μM and 20 μM slightly reduced RGC density to 1731.5 ± 789.7 and 1699.8 ± 588.7 cells/mm^2^, respectively (Figure 6g).

### 3.7. PLX5622 Prevents Pressure-Induced Apoptosis and Retinal Degeneration

**Figure 7:**
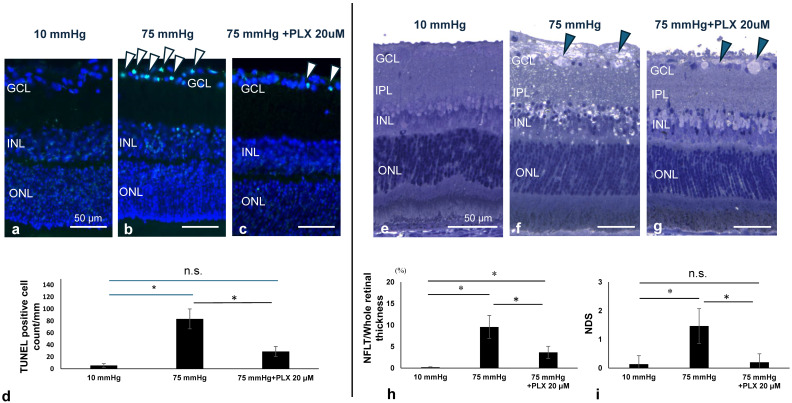
(**a**–**c**) TUNEL staining. At 10 mmHg (**a**), few TUNEL stain-positive cells were observed. At 75 mmHg (**b**), apoptosis (arrowheads) was observed in the RGC layer, which was suppressed by 20 μM PLX5622 (**c**). (**d**) Number of apoptosis-positive cells under each condition (*n* = 6 in each experiment, Tukey’s multivariate comparison, * *p* < 0.05). (**e**–**g**) Light micrographs of semi-thin sections: (**e**) 10 mmHg; (**f**) 75 mmHg (Note axonal swelling of the NFL (arrowheads) and injury to the GCL, IPL, and INL); (**g**) Administration of PLX5622 suppressed injury to the GCL, IPL, INL and OPL. Axonal swelling of the NFL was still present (arrowhead). (**h**,**i**) NFLT vs. RT (%) and NDS under each condition (*n* = 5 in each experiment, Tukey’s multivariate comparison, * *p* < 0.05). “n.s.”: non-significant.

As previously reported [25], TUNEL-positive cells are infrequently observed in the ganglion cell layer (GCL) at 10 mmHg (Figure 7a) but markedly increased at 75 mmHg (Figure 7b). We found that PLX5622 prevented RGC apoptosis at 75 mmHg (Figure 7c). Figure 7d shows the number of TUNEL-positive cells in the GCL in each condition (also see Supporting Figure S2).

Retinas exhibited normal morphological appearance when incubated at 10 mmHg for 24 h (Figure 7e) but showed axonal swelling in the nerve fiber layer (NFL) as well as damage in the GCL, inner plexiform layer (IPL), and inner nuclear layer (INL) at 75 mmHg (Figure 7f), as we have reported previously [25]. Administration of PLX5622 markedly attenuated damage in the GCL, IPL, and INL, while axonal swelling by high pressure remained (Figure 7g). Figure 7h,i show measures of nerve fiber layer thickness (NFLT) and NDS in retinas under each condition, respectively. Quantitative analysis showed that the number of TUNEL-positive cells increased from 9.44 ± 6.78 at 10 mmHg to 80.56 ± 14.33 at 75 mmHg and was reduced to 23.33 ± 13.33 by PLX5622 treatment (Figure 7d). The thickness of NFLT increased from 0.18 ± 0.11 at 10 mmHg to 9.56 ± 2.66 at 75 mmHg and decreased to 3.64 ± 1.42 with PLX5622 (Figure 7h). The NDS increased from 0.13 ± 0.30 at 10 mmHg to 1.47 ± 0.61 at 75 mmHg and was reduced to 0.20 ± 0.30 with PLX5622 (Figure 7i).

### 3.8. Effects of PLX5622 on NLRP3 Inflammasome Pathway Markers

**Figure 8:**
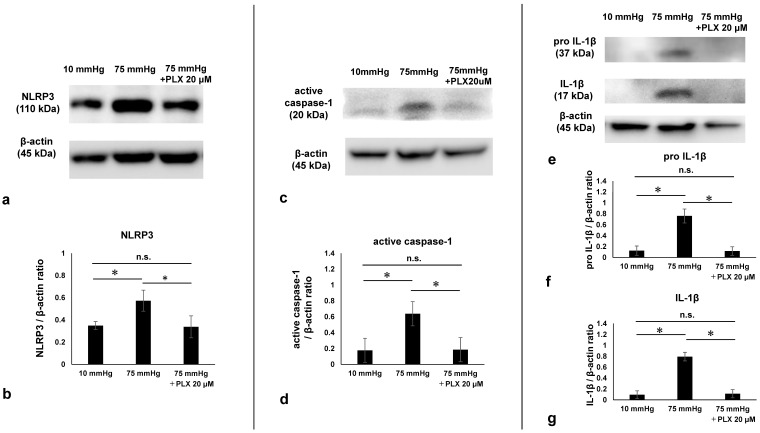
Western blotting of changes in expression of NLRP3, active caspase-1, pro IL-1β, and IL-1β before and after PLX5622 administration. (**a**) Representative immunoblots for NLRP3 and actin. (**b**) Relative densitometry analysis of NLRP3 expression (*n* = 5 in each experiment, Tukey’s multiple comparison test, * *p* < 0.05). (**c**) Representative immunoblots for active caspase-1 and actin. (**d**) Relative densitometry analysis of active caspase-1 expression (*n* = 5 in each experiment, Tukey’s multiple comparison test, * *p* < 0.05). (**e**) Representative immunoblots for pro IL-1β, IL-1β, and actin. (**f**,**g**) Relative densitometry analysis of pro IL-1β (**f**) and IL-1β expression (**g**) (*n* = 6 in each experiment, Tukey’s multiple comparison test, * *p* < 0.05). The samples shown were derived from separate gels and experiments conducted on different days. All blots were processed using identical protocols. “n.s.”: non-significant.

Western Blot analysis demonstrated that administration of 20 μM PLX5622 significantly decreased NLRP3 expression compared to drug-free pressure elevation (Figure 8a). Figure 8b shows the density of NLRP3 in retinas in each condition. Active caspase-1 exhibited multiple bands, as in previous reports [29,30]. We identified and analyzed a specific band of active caspase based on molecular weight at approximately 20 kDa. Pressure-induced increases in expression of active caspase-1 were also decreased by administration of PLX5622 (Figure 8c). Figure 8d shows the density of active caspase-1 in retinas in each condition. Consistent with observations with NLRP3 and caspase-1, Western Blot analysis demonstrated that administration of PLX5622 significantly decreased pressure-induced changes in the expression pro IL-1β and IL-1β (Figure 8e). Figure 8f,g display the density of IL-1β and pro IL-1β in retinas under each condition. Quantitative analysis showed that NLRP3 expression increased from 0.35 ± 0.036 at 10 mmHg to 0.57 ± 0.095 at 75 mmHg and was reduced to 0.34 ± 0.099 with 20 μM PLX5622 (Figure 8b). Active caspase-1 levels increased from 0.18 ± 0.078 at 10 mmHg to 0.64 ± 0.076 at 75 mmHg and decreased to 0.19 ± 0.122 with PLX5622 (Figure 8d). Expression of pro IL-1β increased from 0.12 ± 0.086 at 10 mmHg to 0.76 ± 0.13 at 75 mmHg and was suppressed to 0.12 ± 0.079 with PLX5622 (Figure 8f). IL-1β expression increased from 0.094 ± 0.072 at 10 mmHg to 0.79 ± 0.079 at 75 mmHg and was reduced to 0.11 ± 0.075 with PLX5622 (Figure 8g).

## 4. Discussion

To our knowledge, this is the first paper to demonstrate that a CSF-1R inhibitor, PLX5622, can protect RGCs during IOP elevation when directly and acutely administered to the retina to deplete microglia. In the present study, we used a rat ex vivo acute glaucoma model in which dissected eyecups are incubated under hydrostatic pressure for 24 h. The hydrostatic pressure was adjusted to simulate conditions in the normal retina (10 mmHg) and conditions that can occur during an AAC (75 mmHg). The advantages of this model make it possible to examine direct effects of pressure loading on microglia and neuroinflammation without invasion of circulating macrophages [25].

We were interested in determining whether microglia in isolated retinas have the ability to proliferate or activate and produce proinflammatory cytokines like IL-1β in a hyperbaric condition mimicking an AAC. Sholl analysis of Iba-1-stained cells showed retraction of microglial ramifications under the hyperbaric condition. Immunohistochemistry using anti-CD68 antibody (M1 marker) revealed that pressure elevation induced proliferation of a neurotoxic form of M1-type microglia. Western blotting demonstrated that pressure-induced IL-1β production occurred via activation of the TLR4–NLRP3 inflammasome cascade, resulting in RGC death. Thus, we hypothesize that inhibition of neuroinflammatory pathways associated with microglial activation may be neuroprotective.

It has been demonstrated consistently that suppression of microglial activation protects RGCs in various glaucoma models including optic nerve crush or transection [31,32], ocular hypertension [33,34], and the DBA/2J mouse genetic model [35]. Microglia specifically express CSF-1R on their surface membrane [36]. CSF-1 is a growth factor involved in the proliferation, differentiation, and survival of microglia via CSF-1R [37,38]. PLX5622 is a highly selective CSF-1R inhibitor and eliminates microglia rapidly and sustainably [39]. A previous study indicates that prolonged in vivo treatment with PLX5622 ablates microglia almost completely in the retina [40], and pharmacological depletion of microglia with PLX5622 is neuroprotective in different animal models by reducing neuroinflammation. In the optic nerve crush model, mice treated with PLX5622 in their diet for 3 weeks before nerve crush and maintained on their diet until sacrifice, showed microglial depletion and attenuation of loss of RGCs and axonal damage [41]. In a mouse model of retinal excitotoxicity induced by intravitreal NMDA injection, pharmacological ablation of microglia with PLX5622-containing diet from 7 days before to 7 days after NMDA injection protects RGCs [42]. In these two in vivo animal models, microglia were almost completely deleted. By contrast, PLX5622 resulted in exaggerated RGC loss 4 weeks after intracameral injection of microbeads in a mouse in vivo glaucoma model [43]. It should be noted that in this latter study, PLX5622 was administered in the diet for 3 weeks before microbead injection, and the PLX5622 diet was not continued throughout the experimental period (for 4 weeks after the microbead injection). Under such experimental conditions, it is likely that microglia repopulate by self-renewal to restore baseline levels [44] after initial pharmacological depletion. Microglial repopulation also can arise from infiltration by peripheral macrophages [45,46]. Recently, macrophage recruitment has been implicated in glaucoma pathogenesis [13,47]. Consistently, significant elevation of serum macrophage chemoattractant protein-1 (MCP-1), a key chemotactic factor in monocyte recruitment often implicated in initiating inflammation [48,49], is associated with visual field loss in glaucoma patients [48]. If this is the case, macrophages infiltrating into the retina after depletion with PLX5622 administration might induce exaggerated RGC loss after intracameral injection of microbeads.

In the present study, we found that there is no effect of PLX5622 on control numbers of microglia. By contrast, PLX5622 reduced microglia by about 50% in the high-pressure condition, attenuating high-pressure-induced increases back to near baseline (10 mmHg) values. The method of dissolving PLX5622 in the incubation medium is similar to how the drug would be administered by intravitreal injection in vivo and raises the possibility that PLX5622 may be effective when administered as a one-time intravitreous injection.

TLR4, which is expressed on the plasma membrane of microglia, is a trigger for neuroinflammation [50]. With regard to TLR4 and glaucoma, it has been reported that a single nucleotide polymorphism in the TLR4 gene is associated with normal tension glaucoma and primary open-angle glaucoma [51]. To date, the details of the molecular signals associated with stress and neuronal injury that cause inflammation in glaucoma remain unclear. One possibility is that damage-associated molecular patterns (DAMPs) released by stressed RGCs or astroglia in the optic nerve head may trigger these inflammatory responses [52,53]. After binding to TLR4, DAMPs activate the NLRP3 inflammasome cascade [54], which activates caspase-1 and produces the proinflammatory cytokine IL-1β. IL-1β is reported to be increased in the blood of glaucoma patients, causing neurotoxic inflammation that leads to axonal degeneration and retinal RGC death [55].

These results suggest that TAK-242, a specific inhibitor of TLR4, may also have potential to protect RGCs in glaucoma. In fact, TAK-242 has been shown to protect RGCs when administered intravitreally following optic nerve crush, a type of injury that damages axons akin to glaucoma [56]. Consistent with this, the present study demonstrated that administration of TAK-242 inhibited pressure-induced increases in NLRP3, indicating that the TLR4 signaling pathway contributes to the pathogenesis of pressure-induced retinal injury. The possible beneficiary actions of TAK-242 against high-pressure mediated retinal damage should be carefully determined in a future study.

Other microglia-modifying treatments include minocycline, a tetracycline derivative known to protect RGCs via suppression of microglial activation in retinal ischemia models [57,58] and in the DBA/2J mouse model of glaucoma [35]. Pharmacologically, the TLR4/NF-κB signaling pathway is also inhibited by minocycline [59], and minocycline upregulates pro-survival genes in glaucoma [60]. Compared to PLX5622, minocycline may have diverse RGC-protective actions besides effects on microglial activation. Taken together, these data suggest that regulation of microglial function could be a rational therapeutic approach to preserve RGCs from the effects of inflammatory cytokines in glaucoma.

Results from our histological studies indicate that PLX5622 suppressed RGC death, while pressure-induced axonal swelling in the NFL was not altered. In a previous study [57], we found that pressure-loading depresses the expression of two key glial proteins, the glutamate transporter, GLAST (EAAT1), and the glutamate-catalyzing enzyme, glutamine synthetase (GS), both of which are required for regulating extracellular glutamate levels and glutamate metabolism. Furthermore, axonal swelling is induced by administration of TFB-TBOA, a potent inhibitor of glutamate transporters, and MSO, a specific GS inhibitor even at 10 mmHg [61], indicating the possibility that axonal swelling is associated with impairment of glutamate metabolism. In long-term cultured spinal motor neurons, activation of neuronal glutamate receptors induces axonal swelling and accumulation of cytoskeletal proteins in the distal segments of axons [62]. Consistent with this, NMDA-induced axonal swelling has been reported in optic nerve sections [63]. Similar events occurring in axons could contribute to the findings observed in the present study. If axonal swelling is the initial event induced by pressure elevation followed by neuronal injury, these findings indicate that PLX5622 can only inhibit retinal injury subsequent to axonal swelling but not axonal swelling itself, at least under the acute conditions we studied. It remains to be determined whether more complete suppression of microglia with longer PLX5622 treatment also affects RGC survival. If retinal axonal swelling is mainly pressure-dependent, IOP reduction should be effective to inhibit axonal swelling. Because PLX5622 is unlikely to alter IOP, it may be more appropriate therapeutically if PLX5622 is combined with treatments that control IOP.

Taken together, our findings indicate that activation of neuroinflammatory pathways associated with activated microglia has important roles in retinal injury under hyperbaric conditions. If activated microglia play a dominant role in pressure-induced neuronal damage, inhibition of microglia using PLX5622 may serve as a potential therapeutic to preserve RGCs. Future studies should address whether neuroprotection by PLX5622 is also observed in acute in vivo glaucoma models.

## Data Availability

The datasets generated and analyzed during the current study are available in the Zenodo repository: https://zenodo.org/records/15873244. However, restrictions apply to a part of the datasets. The datasets presented in this article are part of an ongoing study. Requests to access the relevant data sets, should be directed to the corresponding author(s).

## Supplementary Materials

The following supporting information can be downloaded at: https://www.mdpi.com/article/10.3390/biomedicines13071670/s1, Figure S1: Light micrographs of retinas exposed to elevated pressure (75 mmHg) for 0 h (a), 12 h (b), and 24 h (c). (d) NDS after incubations at 75 mmHg for 0 h, 12 h, or 24 h in a time-dependent manner; Figure S2: Representative fluorescence images of cryosections stained for TUNEL (green) and RBPMS (red).

## Abbreviations

The following abbreviations are used in this manuscript:

RGC	Retinal ganglion cells
AAC	Acute angle-closure attack
IOP	Intraocular pressure
IL	Interleukin
NLRP3	Nucleotide-binding and oligomerization domain, leucine-rich repeat-, and pyrin domain-containing protein 3
NLR	NOD-like receptor
PRR	Pattern recognition receptor
TLR	Toll-like receptor
I/R	Ischemia/reperfusion
CSF-1R	Colony stimulating factor 1 receptor
aCSF	Artificial cerebrospinal fluid
SD rat	Sprague Dawley rat
RBPMS	mRNA processing factor
Iba-1	Ionizing calcium-binding adaptor molecule 1
NFLT	Nerve fiber layer thickness
NDS	Neuronal damage score
INL	Inner nuclear layer
IPL	Inner plexiform layer
GCL	Ganglion cell layer
