# Supplementary material for: Involvement of Microglia in Retinal Ganglion Cell Injury Induced by IOP Elevation in a Rat Ex Vivo Acute Glaucoma Model
Source: Biomedicines. 2025 Jul 8;13(7):1670. doi: 10.3390/biomedicines13071670 (PMC12292869; doi:10.3390/biomedicines13071670)
Supplement: Supplementary file 1 [file biomedicines-13-01670-s001.zip › biomedicines-3670313-supplementary.pdf]

Supporting Figure S1

After incubations at 75 mmHg for 0 h, 12 h, and 24 h, eyecups were fixed in 1% paraformaldehyde and 1.5% glutaraldehyde-0.1 M phosphate buffer overnight at 4 °C. The fixed eye cups were rinsed in 0.1 M phosphate buffer and placed in 1% buffered osmium tetroxide for 60 minutes. Specimens were dehydrated with alcohol, embedded in Epon 812 resin (TAAB Laboratories; Aldermaston, Berks, UK), and cut into 1-μm-thick sections for light microscopy. The sections were then stained with toluidine blue and evaluated by light microscopy.

Retinas exhibited no remarkable changes after incubations at 75 mm Hg for 0 h (Figure S1a) and 12 h (Figure S1b). After 24 h at 75 mm Hg, axonal swelling was prominent, but the other layers remained almost intact (Figure S1c). Neuronal damage score (NDS) was summarized in Figure S1d.

In our preliminary experiments, unspecific neurodegeneration occurs even in control retinal samples when incubated for 48 hours. Therefore, we considered 24 hours as the most appropriate incubation time at 75 mmHg.

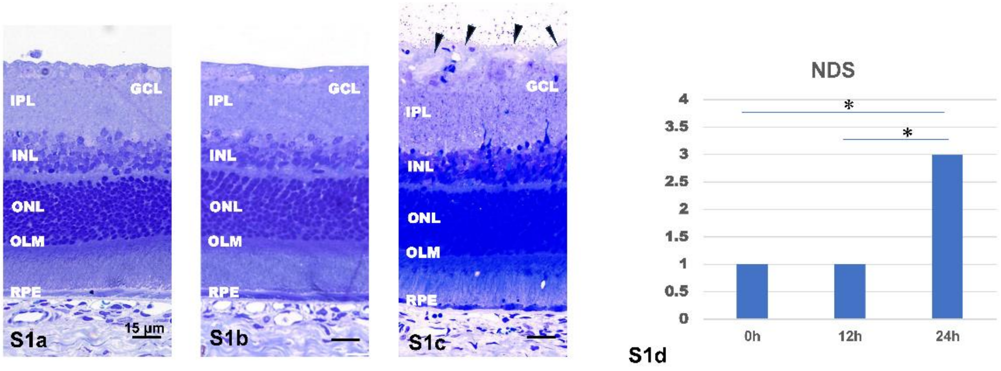

Supporting Figure S1. a–c. Light micrographs of retinas exposed to elevated pressure (75 mmHg) for 0 h (**a**), 12 h (**b**), and 24 h (**c**). (**a, b**) The retinas exhibited normal morphology at 75 mmHg for 0 h (**a**) and 12 h (**b**). Swollen axons were not detected in the nerve fiber layers. (**c**) Exposure to 75 mm Hg for 24 h revealed axonal swelling (arrowheads) in the NFL. (**d**) NDS after incubations at 75 mmHg for 0 h, 12 h, or 24 h in a time-dependent manner. Statistical differences were analyzed using Tukey's multiple comparison test (n = 3 in each experiment). * $p < 0.05$.

Supporting Figure S2

To confirm whether the apoptotic cells detected by TUNEL staining in the ganglion cell layer were indeed RGCs, we performed double immunofluorescence staining for TUNEL and RBPMS. Although TUNEL-positive signals in this region were previously interpreted as RGC apoptosis, the presence of other cell types warranted verification by co-localization analysis.

Materials and methods

A number of cryosections were subjected to TUNEL staining using the ApopTag Fluorescein In Situ Apoptosis Detection Kit (Sigma-Aldrich, St. Louis, MO) according to the manufacturer's instructions. After staining, the sections were washed three times with Tween-PBS (Tw-PBS) for 5 minutes each. The sections were then incubated in 0.5% Triton X-100 in Tw-PBS for 10 minutes at room temperature, followed by three additional washes in Tw-PBS. Non-specific binding was blocked by incubation with 10% donkey serum in Tw-PBS for 1 hour at room temperature. After blocking, the sections were incubated overnight at 4 °C with rabbit anti-RBPMS antibody (1:1000, Cat#ab152101, Abcam, Waltham, MA). On the following day, sections were washed three times with Tw-PBS and incubated for 1 hour at room temperature with goat anti-rabbit IgG (H&L) cross-adsorbed secondary antibody conjugated to Alexa Fluor™ 488 (1:1000, Cat#A-11008, Invitrogen Corp, Carlsbad, CA, USA). After a final series of washes in Tw-PBS (three times, 5 minutes each), the sections were mounted with Vectashield Mounting Medium containing DAPI (Cat#H-1200, Vector Laboratories Inc., Newark, CA, USA). Fluorescent images were acquired using a fluorescence microscope (BZ-800X, Keyence, Japan).

.

Results and Discussion

As shown in Supporting Figure S2, TUNEL-positive cells were not observed at 10 mmHg (Supporting Figure S2a). At 75 mmHg (Supporting Figure S2b), a number of double labelled TUNEL-positive/RBPMS-positive RGCs were observed in the GCL. In contrast, in the 75 mmHg + PLX5622 group (Supporting Figure S2c), there were only few TUNEL-positive/ RBPMS positive RGCs.

These findings indicate that the apoptotic cells detected by TUNEL staining in the GCL were indeed RGCs, which is mitigated by PLX5622 treatment.

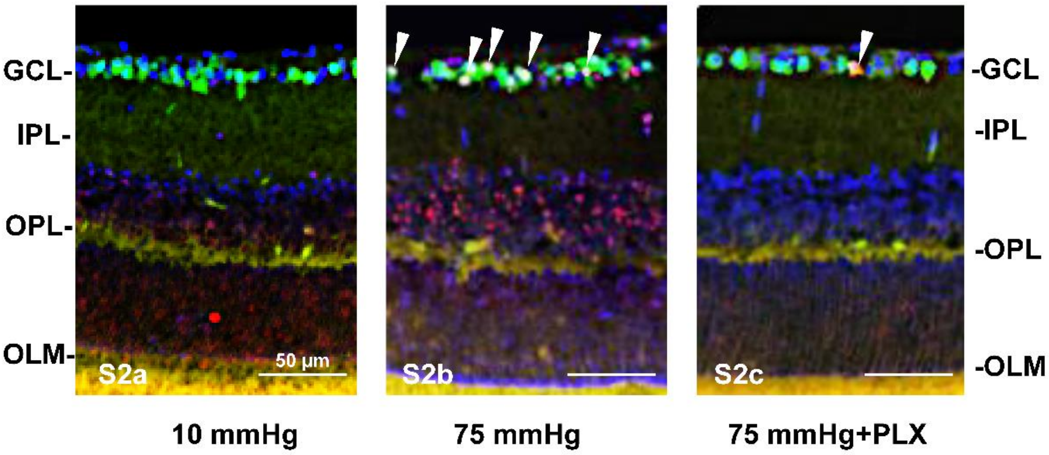

Supporting Figure S2. Representative fluorescence images of cryosections stained for TUNEL (green) and RBPMS (red). Co-localization of TUNEL/RBPMS is visualized in a merged image. **a.** At 10 mmHg, few TUNEL-positive cells were detected. **b.** At 75 mmHg, TUNEL-positive/RBPMS-positive RGCs increased. **c.** Apoptotic RGCs was markedly reduced by treatment with 20 µM PLX5622.
